# Composite Micro-Nanoarchitectonics of MMT-SiO_2_: Space Charge Characteristics under Tensile State

**DOI:** 10.3390/polym13244354

**Published:** 2021-12-13

**Authors:** Hongtao Jiang, Junguo Gao, Xiaohong Zhang, Ning Guo

**Affiliations:** Key Laboratory of Engineering Dielectrics and Its Application, Ministry of Education, Harbin University of Science and Technology, Harbin 150080, China; jianghongtao012@163.com

**Keywords:** mechanical stretch, micro-nano composites, crystalline morphology, charge transport, space charge

## Abstract

Low density polyethylene (LDPE) is a good insulating material which is widely used in cable materials due to its excellent insulation and processability. However, in the DC high voltage environment, pure polyethylene materials still face many problems, the most serious of which is space charge accumulation. The cable will inevitably be subjected to tensile stress during production, installation and operation. Therefore, it is of great significance to study the effect of stretching on the microstructure and space charge characteristics for polymers and their composites. In this paper, MMT/LDPE micro-composites, SiO_2_/LDPE nano-composites and MMT-SiO_2_/LDPE micro-nano-composites were prepared by melt blending. Mechanical stretching was carried out on pure LDPE materials and the above three kinds of composite materials. Each material was stretched according to four stretching ratios, which are 0%, 5%, 10% and 20%. The crystal morphology was observed by polarizing microscope (PLM), the crystallization perfection was tested by differential scanning calorimetry (DSC), and the space charge distribution inside each sample was measured by pulsed electro-acoustic (PEA) method. At the same time, the average charge density and apparent charge mobility for samples during depolarization were calculated and analyzed. The experimental results show that when the pure low density polyethylene sample is not stretched, its crystal structure is loose. Tensile stress can make the loose molecular chains align in LDPE and improve its crystalline structure, which is helpful to restrain the accumulation of space charge inside the sample. For MMT/LDPE, SiO_2_/LDPE and MMT-SiO_2_/LDPE composites, their internal crystal structure is compact. Stretching will destroy their original crystal structure at first, and then disorder molecular chains inside the three composite materials. With the increase of stretching ratio, the molecular chains begin to orient along the direction of force, the crystallization tends to be perfect gradually, and the space charge accumulation in samples also decreases. From the calculation results of apparent charge mobility for each sample, with the increase of stretching ratio, the trap depth and trap density inside samples firstly increased and then decreased.

## 1. Introduction

Since polymer materials were born, they have always been adopted in many fields of industry and life. Among them, crystalline polymers occupy about two-thirds of the market, which shows their important position in national production. In the power sector, polyethylene has been widely used in high voltage DC cable insulation result from its excellent electrical insulation performance [1]. However, in practical applications such as production, installation and operation, power cables are inevitably affected by external tensile forces [2,3,4]. For example, when the cable material is extruded, tensile stress will occur due to the different cooling rates of the inner layer and outer layers. During installation, the cables may bend. Different thermal expansion coefficient of cable insulation and conductor in operation will also cause the cable to bear stress. Additionally, when the cable is working, the load is not always constant. The variation of the cable load will lead to the variation of the working environment temperature. Therefore, the morphology of the insulating polymer used in cable is constantly changing during the use. These inevitable mechanical stretching will change the local shape and structure in cables and the distribution of internal traps, which will further affect the electrical performance and safe operation of the cable.

Under normal circumstances, the polymer is in a state of equilibrium and its internal structure is uniform network. When subjected to tensile stress, the arrangement of molecular chains in both amorphous and crystalline regions will change [5,6]. This will affect the crystalline structure of the polymer to a certain extent. Meanwhile, the properties of semi-crystalline polymers are closely related to the aggregate structure for the polymer itself, such as crystallinity, crystal size, grain boundaries and internal defects [7,8]. Then, the change in the microstructure caused by tensile stress could lead to changes in the macroscopic electrical properties of materials. Therefore, it is of great significance to explore the relationship between microstructure and electrical properties of composite materials after tensile stress.

Under the condition of DC high voltage, the injection and accumulation of space charge in polyethylene may be intensified [9,10], which will make the local electric field intensity too high, shorten the aging time, and limit the application of high voltage DC cable made of polyethylene in an engineering sense. Yin pointed out that using different kinds of nano-powders to fill the polymer will make the performance of the composite appear extreme value at a certain point. Because of the different functional groups, quantity and surface thickness of different particles, the particles interact with the matrix and also adsorb each other, thus presenting synergistic effect [11]. When micron-size particles and nano-particles are added to the polymer, besides the type and number of functional groups and the thickness of surface layer, the shape and size of particles will be further introduced. The interaction between micro and nano particles and their interaction with the matrix material will affect the crystallization behavior of the composites, and then affect the macroscopic properties. At present, many experts and scholars have achieved the improvement of matrix performance by adding micro and nano particles in the matrix [12,13]. For example, Zhou et al. used layered micro-nano material zirconium phosphate as micro-nano carrier, and inserted octadecyl triphenyl phosphine bromide between ZrP sheets by ion exchange method, and then loaded cuprous oxide on the surface of ZrP. A kind of micro/nano sheet structural material Cu_2_O@OZrP was constructed. When this sheet material was added to the matrix polyethylene terephthalate with the content of 0.2%, the composite showed good mechanical properties and antibacterial properties [14]. Wang et al. synthesized MoP/CNTs microspheres by spray drying and phosphating process, and embedded carbon nanotubes (CNTs) into MoP/CNTs microspheres, which not only improved the conductivity of the composites, but also alleviated the volume change during cycling [15]. Dai et al. prepared micro-nano composite materials with epoxy as matrix, aln as micron additive particles and Al_2_O_3_ as nano additive particles. It was found that when 20 wt% micro-AlN and 1 wt% nano-Al_2_O_3_ were added, the composite materials had the smallest space charge accumulation, higher thermal conductivity and better thermal stability. When the content of micro-particles is increased to 50%, the thermal conductivity of the composites is obviously improved [16].

Now that micro-nano composite technology has been widely used, what about the space charge characteristics of micro-nano composite materials under tension? In this paper, low density polyethylene is used as matrix, layered montmorillonite is selected as micron particles, and silica is selected as nano particles. The materials needed for experiments are prepared by melt blending technology. Considering the actual tensile stress of cables, the tensile ratio of 0%, 5%, 10% and 20% is selected to explore the effect of tensile stress on the crystalline structure and space charge characteristics of composite materials.

## 2. Experimental Preparation and Testing

### 2.1. Experimental Materials

The SiO_2_ particles and silane coupling agent KH570 selected in the experiment were purchased from Beijing Deke Daojin Science and Technology (Beijing, China). The size of SiO_2_ particles is 30 nm, the purity is 99.9%, the specific surface area is 200 m^2^/g, the bulk density was 0.08 g/cm^3^, and the real density was 2.2 g/cm^3^. The montmorillonite (MMT) was purchased from Qinghe Chemical Plant (Zhangjiakou, China). Low density polyethylene (LDPE), purchased from Jinshan Petrochemical Company (Shanghai, China), has a density of 0.924 g/cm^3^ and a melting index of 2 ± 0.3 g/(10 min·2.16 kg).

### 2.2. Instrumentation and Equipments

RM-200A torque rheometer (Hapu electrical technology limited liability company, Harbin, China), CMT4103 universal testing machine (MTS systems corporation, Minneapolis, MN, USA), LeicaDM2500 polarizing microscope (PLM, Leica Microsystems, Wetzlar, Germany), DSC-1 differential scanning calorimeter (DSC, Mettler Toledo, Zurich, Switzerland), Pulsed electro-acoustic space charge test system (Shanghai Jiao Tong University, Shanghai, China).

### 2.3. Preparation of Composite Materials

**Figure 1:**
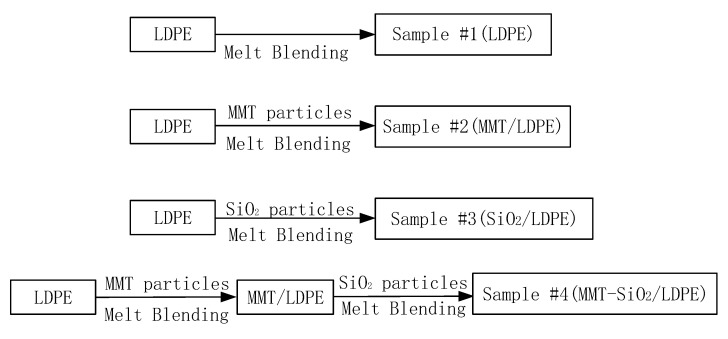
Material preparation process.

SiO_2_ particles treated by KH570 coupling agent, MMT particles modified by organic intercalation of KH570 and octadecane trimethylammonium chloride, both of them were fused and blended with LDPE matrix using a torque rheometer. The mixing temperature was set at 140 °C, the mixing time was set at 20 min, and the addition content of micro and nano particles was 1 wt%. Then, the prepared pure LDPE, micron composite, nano composite and micro-nano composite materials were pressed into the film on the flat plate vulcanizing machine. The temperature was 140 °C, the pressure was increased to 15 MPa step by step, the time was 20 min, and at 15 MPa, the circulating water was cooled for 3 min, and four kinds of composite materials were prepared. The preparation process of the four composite materials is shown in Figure 1.

Each composite material sample was divided into four parts, which are stretched by 0%, 5%, 10% and 20% with a universal testing machine. The tensile rate is 4 mm/min. When the stretching ratio reached the corresponding ratio, the universal testing machine stopped stretching and kept the stretching state for 24 h to obtain the samples required for the experiment in this paper. The sample number information is shown in Table 1.

**Figure 2:**
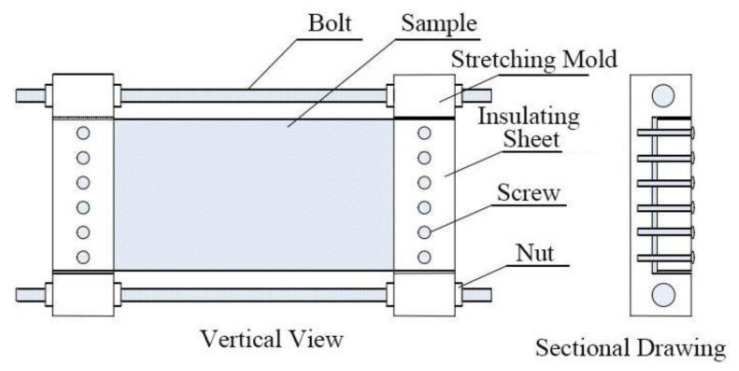
Tensile fixing device.

It is worth noting that before testing the performance of each sample, the fixture as shown in Figure 2 made by the laboratory should be used to fix the tensile state of each sample and then remove it.

### 2.4. Observation of Crystallization Behavior

In order to explore the influence of tensile stress on the internal crystal morphology of the sample, a polarizing microscope is needed. For getting the crystal morphology pictures of each sample clearly, it is necessary to etch all materials with 5% potassium permanganate and concentrated sulfuric acid solution in advance. After etching, the samples were put into the ultrasonic cleaning machine for cleaning. Then the crystal morphology of each sample was observed under a polarizing microscope.

### 2.5. Crystallinity Test

The crystallinity of each sample was tested by differential scanning calorimetry. About 8 mg of each sample was sampled and sealed in a small aluminum crucible, and then placed in a test instrument for measurement. The temperature range of the measurement was set to 25~150 °C, and the temperature rise and fall rate was set to 10 °C/min. The whole experiment process was carried out under the protection of liquid nitrogen, and the heat flow curve during the melting process of each sample was recorded.

### 2.6. Space Charge Test

The space charge distribution characteristics of each sample were tested by pulsed electro-acoustic space charge test system independently developed by Shanghai Jiaotong University. The basic principle is that a nanosecond high-voltage narrow pulse wave is injected into the sample to be tested through the electrode. The propagation of high-voltage narrow pulse waves in the sample will produce different disturbances to all kinds of bound charges in the sample, so that all kinds of bound charges produce different degrees of micro displacement, and then the acoustic wave propagates to the opposite electrode. The acoustic signal is collected and processed by PVDF piezoelectric sensor, and the acoustic pulse signal is converted into the corresponding electrical pulse. Then, by processing and analyzing these electrical pulse signals with computer software, the space charge distribution at different positions in the sample can be obtained.

**Figure 3:**
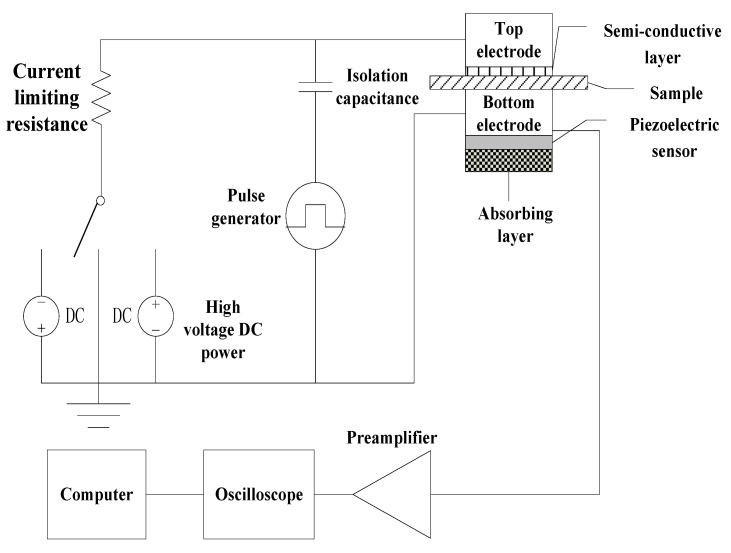
Test system of pulsed electro-acoustic method.

The structure of the testing device is shown in Figure 3, which includes: adjustable DC power supply 0~20 kV; pulse generator, amplitude is 1.0 kV, width is 30 ns; PVDF thin film piezoelectric sensor with thickness of 30 μm; 400 MHz preamplifier, 400 MHz digital oscilloscope and computer processing system. After the sample was put into the test system, the sound speed selection button was set to LDPE option, the pulse voltage was set to 400 V, and the test method was selected as reference measurement. The reference waveform was collected with 3 kV/mm electric field polarizing sample for 5 min. After the reference waveform test was completed, the test method was changed to pressure measurement, and the sample was polarized for 30 min under the field intensity of 10 kV/mm, 20 kV/mm and 40 kV/mm, respectively. All the tests were completed, the system’s own data recovery software was used to restore the experimental data.

## 3. Result and Discussion

### 3.1. Observation of Crystalline Morphology for Composites

**Figure 4:**
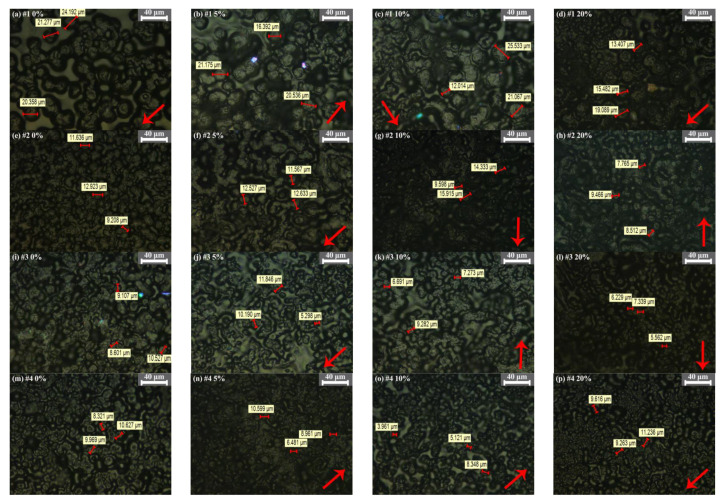
The crystalline morphology of all samples (the red arrow indicates the direction of tensile stress). ((**a**–**d**) are the crystalline morphology of sample 1 at 0%, 5%, 10% and 20% tensile ratios respectively; (**e**–**h**) are the crystalline morphology of sample 2 at 0%, 5%, 10% and 20% tensile ratios respectively; (**i**–**l**) are the crystalline morphology of sample 3 at 0%, 5%, 10% and 20% tensile ratios respectively; (**m**–**p**) are the crystalline morphology of sample 4 at 0%, 5%, 10% and 20% tensile ratios respectively).

Figure 4 is the crystal morphology of each sample observed under polarizing microscope. It can be seen that with the increase of tensile ratio, the crystal cell spacing of sample 1 becomes smaller and smaller, and the crystal structure develops in a close direction. When the tensile ratio reaches 10%, there are fine grains. When the tensile ratio reaches 20%, fine grains and large grains are arranged closely. The crystal structure of sample 2, 3 and 4 is relatively tight without stretching. After 5% stretching, the crystal structure of these three composites began to loosen, and the crystal cell gap gradually increased. Sample 4 has fine grains when the cell spacing increased. When the tensile ratio was 10%, the crystal cell of samples 2, 3 and 4 have a trend of arranging along one direction, but the crystal morphology is still loosely arranged. When the tensile ratio reached 20%, the crystal structure of each sample began to become dense, and the crystal cells appears orientation along the stress direction.

### 3.2. Dsc Test of Composite Materials

**Figure 5:**
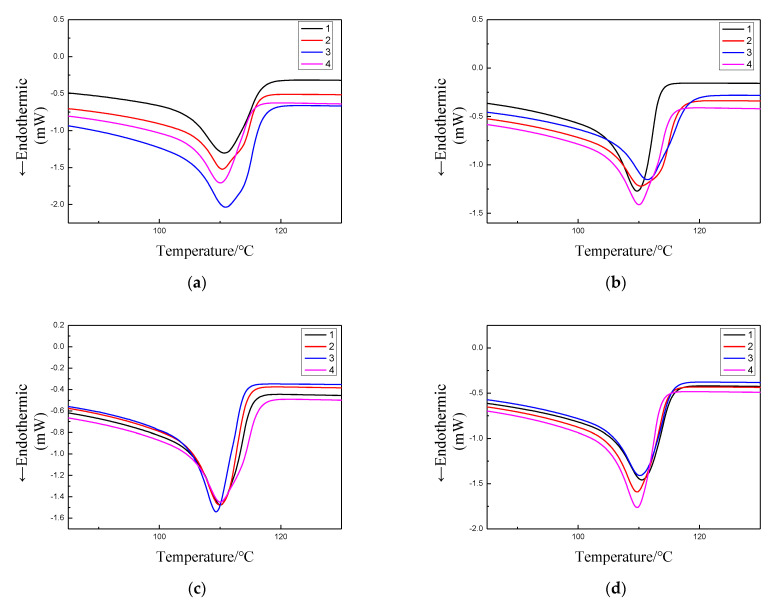
DSC curves of each sample. (**a**) Tensile rate 0%; (**b**) Tensile rate 5%; (**c**) Tensile rate 10%; (**d**) Tensile rate 20%.

Differential scanning calorimetry (DSC) is a method to compare the sample to be measured with the reference material, draw the curve of heat flow changing with time or temperature, and then analyze the crystallization parameters of the sample. The obtained curves usually take in Celsius temperature T or time t as the abscissa and the heat flow as the ordinate. The test results are shown in Figure 5. The melting peak temperature can be directly obtained from the instrument, as shown in Table 2.

The melting enthalpy of each composite during melting can be calculated by Formula (1) [17].

(1) $$∆H_{m}=60\int_{T_{i}}^{T_{f}}\frac{Q_{H\left(T\right)}}{B}dT$$

where $T_{i}$ and $T_{f}$ are the starting temperature and ending temperature of the melting peak. $Q_{H\left(T\right)}$ is the differential heating/cooling rate (W/g). B is the rise/fall rate, and the calculation results of $∆H_{m}$ are shown in Table 2.

The crystallinity of each sample can be calculated according to the Formula (2), and the calculation results are shown in Table 2 [18].

(2) $$X_{c}=\frac{∆H_{m}}{∆H_{0}}\times 100\%$$

where *H*_0_ = 293.6 J·g^−1^ (LDPE crystallization melting enthalpy). The melting temperature, crystallinity and melting heat of complete crystallization for all samples are summarized in Table 2.

When the structure of polyethylene changes from disordered state to ordered crystalline state, it needs to be folded into lamellae through molecular chains, and then the lamellae expand and accumulate to form a large-size spherical crystalline structure [19]. According to the results in Figure 4 and Table 2, it can be found that the crystallinity of sample 1 gradually increases with the increase of stretching ratio. However, the crystallinity of samples 2, 3 and 4 obviously decreases at first and then increases. This corresponds to the experimental results of PLM. The tighter and more regular the crystal structure of the sample, the greater its crystallinity. For sample 1, when it is not stretched, its crystal structure is loose, and stretching makes its crystal structure compact. At the same time, stretching gives energy to the amorphous phase at the edge of the crystal region, and the molecular chains in loose and disordered state tend to align along the same direction under the action of force, which makes this amorphous phase change to crystalline phase, so the crystallinity is improved. For samples 2, 3 and 4, when they were not stretched, their crystal structure was tighter than the matrix. When the stretching ratio was 5%, their tight crystal structure began to loosen and the distance between grains gradually increased, which indicated that the disorder degree of some originally regularly arranged molecular chains increased under the action of tensile stress, which made the crystal structure of samples loose, thus the crystallinity decreased. With the stretching ratio reaching 10%, the molecular chain stretched to chaos begins to rearrange in the direction of force, forming a new crystalline structure, so there will be a phenomenon that the grains begin to have an orientation trend in PLM experiment; When the stretching ratio reaches 20%, the degree of molecular chain regularization is higher, the crystal structure of the three samples becomes compact, and the crystallinity increases accordingly. 

**Figure 6:**
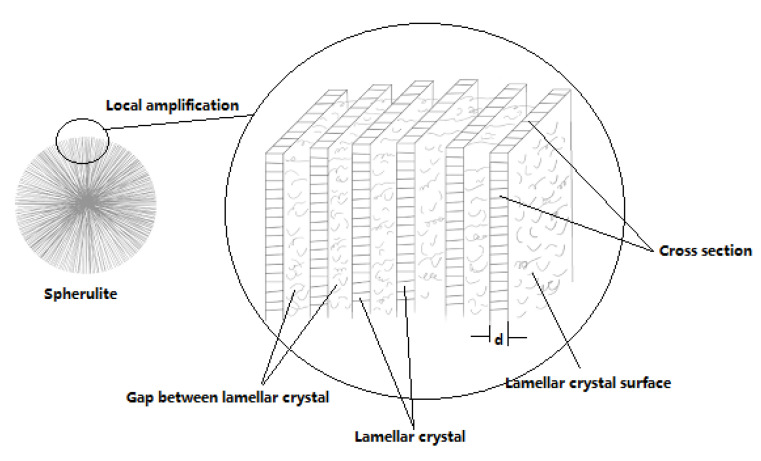
Schematic diagram of spherulite and lamellar crystal structure of polyethylene.

The composition relationship between lamellae and spherulites of polyethylene and the structure of lamellae are shown in Figure 6 [20]. The lamellar crystal thickness *d* is usually used as a parameter to describe the crystallization characteristics. We generally call the plane parallel to *d*, a cross section, and the plane perpendicular to *d*, a lamellar crystal surface. The melting point Tm of polyethylene has a great relationship with the thickness of the lamellar crystal, which satisfies Thomson–Gibbs formula [21,22]:

(3) $$T_{m}=T_{m}^{0}\left(1-\frac{2\sigma_{e}}{d∆H_{h}}\right)$$

According to Formula (3), it can be deduced that:

(4) $$d=\frac{2\sigma_{e}T_{m}^{0}}{∆H_{h}\left(T_{m}^{0}-T_{m}\right)}$$

where, $T_{m}$ is the melting point (K); $T_{m}^{0}$ is the equilibrium melting point (K), which is the melting temperature of infinite thick crystal formed by ideal crystallization of molecular chain with infinite molecular weight, and polyethylene can be taken as 414.6 K; $∆H_{h}$ is the melting enthalpy of the lamellar crystal with infinite thickness, $∆H_{h}=288\times 10^{6}\;J/m^{3}$; $\sigma_{e}$ is the specific free energy of folding surface per unit area, $\sigma_{e}=9.3\times 10^{-2\;}J/m^{2}$; d is the lamellar crystal thickness (*m*).

In order to analyze the influence of tensile action on the lamellar crystal thickness of each composite material, the lamellar crystal thickness was calculated according to Formula (4). The calculation results are shown in Table 3. It can be seen from the data in Table 3 that when the tensile ratio of sample 1 is 5%, the thickness of the lamellae decreases by 2.45% compared with the undrawn sample, but the crystallinity in the DSC result increases by 0.33%. This confirms the previous analysis. Tensile makes the molecular chains of the amorphous phase for pure polyethylene begin to be oriented and arranged regularly. However, the lamellar crystal thickness formed by this effect is not as thick as that formed initially, and the newly formed crystallization is not perfect, thus reducing the overall lamellar crystal thickness. When the tensile ratio increasing to 10% and 20%, the chain segments of the amorphous phase continue to align and the crystallization tends to be perfect, so the thickness and crystallinity of the lamellae are improved, and new fine grains appear in the PLM diagram. For sample 2, the lamellar crystal thickness is always thinning, which may be due to the lamellar structure of MMT particles, prone to orientation under tensile stress. The orientation of MMT particles is more likely to destroy the original crystalline structure of the sample itself, so the crystallinity and lamellar crystal thickness are reduced to varying degrees. From the analysis of PLM images and DSC data, it can be seen that when the stretching ratio reaches 10%, the loose molecular chains begin to have a trend of alignment along the force direction, but the crystallization should be incomplete and belong to the orientation initialization stage, so the crystallinity and lamellar crystal thickness are not very high. When the tensile ratio reaches 20%, more and more disordered molecular chains begin to rearrange into regular lamellae. However, due to the large number of the rearranged lamellae, the lamellae thickness of the sample will be diluted, thus the crystallinity increases and the lamellae thickness decreases. For samples 3 and 4, the crystal structure of the samples is relatively compact when not stretched. It can be seen from the PLM diagram that the distance between the grains of the two samples increases when the tensile ratio is 5%. This also indicates that part of the grains in two samples are condensed, the crystalline region shrinks, and the amorphous region expands, so the crystallinity decreases. When the stretching ratio is further increased, the original crystalline structure is destroyed, and the ordered molecular chains become disordered, resulting in lower crystallinity and thinner lamellar crystal thickness than the samples without stretched. In this series of processes, the crystallinity and lamellar crystal thickness of sample 4 changed very little, which indicated that the crystal structure of micro-nano composites was more solid than other three composites to some extent, and the stress interference resistance was stronger. When the tensile ratio increased to 20%, the disordered molecular chains of sample 3 and 4 began to be neatly arranged, and the grains were obviously arranged along the force direction. The crystallization began to be perfect, the crystallinity increased significantly, and the lamellar crystal thickness also began to increase.

## 4. Space Charge Characteristics of Composite Materials

**Figure 7:**
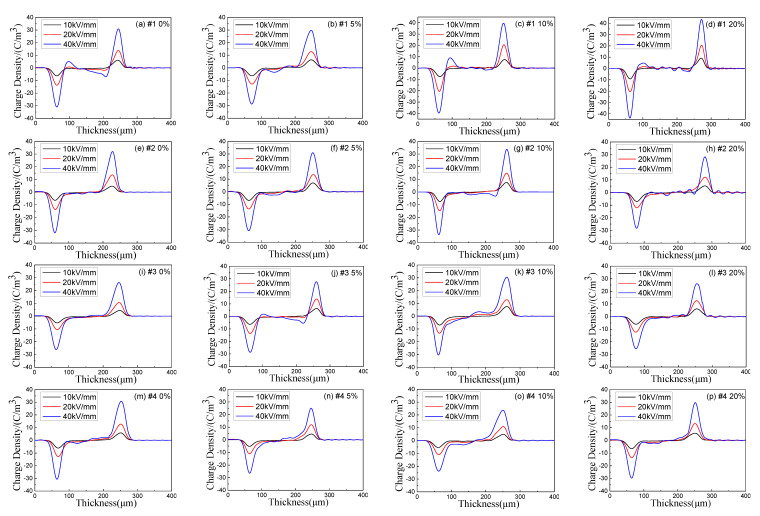
Space charge distribution of composites under polarization electric field after 30 min. ((**a**–**d**) are the space charge characteristic curves of sample 1 under the tensile ratio of 0%, 5%, 10% and 20% respectively; (**e**–**h**) are the space charge characteristic curves of sample 2 under the tensile ratio of 0%, 5%, 10% and 20% respectively; (**i**–**l**) are the space charge characteristic curves of sample 3 under the tensile ratio of 0%, 5%, 10% and 20% respectively; (**m**–**p**) are the space charge characteristic curves of sample 4 under the tensile ratio of 0%, 5%, 10% and 20% respectively).

Figure 7 shows the test results of the internal space charge distribution of each sample immediately polarized for 30 min at 10 kV/mm, 20 kV/mm and 40 kV/mm DC field intensity after tensile treatment of 0%, 5%, 10% and 20%, respectively. It can be seen that no matter the composite materials with added MMT particles, nano SiO_2_ particles, or micro and nano particles together, the space charge accumulation in the sample can be inhibited. When the field intensity is 10 kV/mm, there is no obvious space charge accumulation in each sample. When the field intensity reaches 20 kV/mm, there is a small amount of space charge accumulation in the sample. When the field intensity reaches 40 kV/mm, the space charge accumulation in each sample is obvious. 

**Figure 8:**
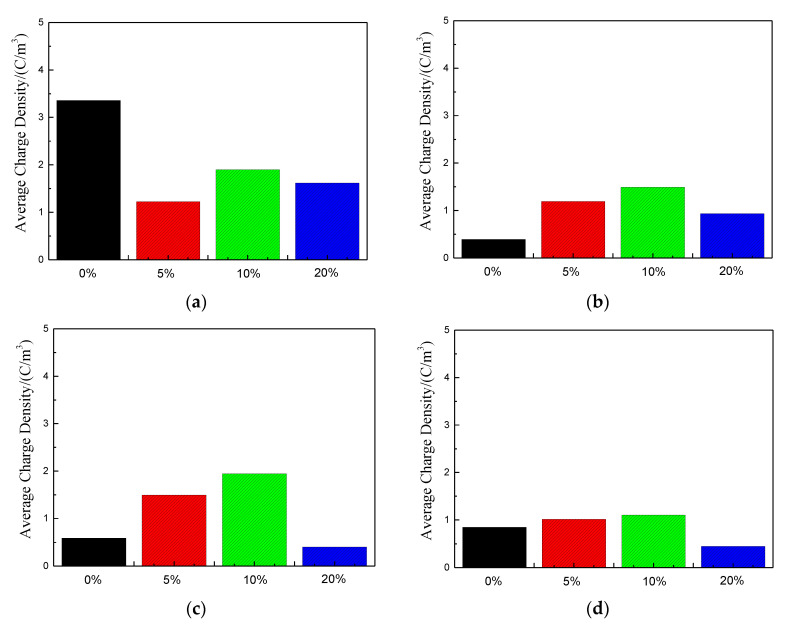
Average charge density for each sample at 40 kV/mm field intensity. (**a**) Sample 1; (**b**) Sample 2; (**c**) Sample 3; (**d**) Sample 4.

In order to further analyze the influence of tension on space charge accumulation, according to Formula (5), the space charge density of different samples is calculated [23,24].

(5) $$Q\left(t,E_{P}\right)=\frac{1}{x_{1}-x_{0}}\int_{x_{0}}^{x_{1}}\left|q_{p}\left(x,t,E_{p}\right)\right|dx$$

where $q_{p}\left(x,t,E_{p}\right)$ is the space charge density inside each sample, usually taking the absolute value; t is the time of applying voltage; $E_{P}$ is the applied electric field intensity value, which is 40 kV/mm. $x_{0}$ and $x_{1}$ usually take the positions where the charge peaks of the lower electrode and the upper electrode is close to the internal zero points of each sample, so as to avoid the influence of induced charges generated at the two electrodes during pressurization. The results are shown in Figure 8.

By observing sample 1, it can be found that under high field intensity, there is much space charge accumulation inside the sample without being stretched. This is because under high field intensity, the internal impurities of LDPE will be ionized, resulting in a large number of anions and cations. The movement rate of these anions and cations is relatively slow, and they are more likely to be trapped, resulting in a large amount of space charge accumulation. When the stretching ratio is 5%, the internal space charge accumulation is significantly reduced. According to PLM and DSC experiment analysis before, this is because the tensile makes its crystalline structure close, but according to the analysis of the lamellar crystal thickness of the before, this kind of crystal structure is not perfect, just start neat arrangement for the molecular chain of amorphous phase, this arrangement may be in a state of relatively loose compared with the molecular chain closely arranged in grain. Additionally, this relatively loose arrangement tending to crystal accounts for a larger proportion than the crystal region, which further increases the scattering effect of carriers, reduces the average free travel of electrons, and is conducive to the neutralization of positive and negative charges, so space charge is not easy to accumulate. When the stretching ratio reaches 10%, the relatively loose molecular chains with a certain orientation begin to become compact, and the crystallization becomes perfect. The lamellar crystal thickness increases, the crystallinity increases, and the chain segments with amorphous phase orientation become compact, which reduces the scattering effect of the loose structure on the carriers, and thus the charge accumulation begins to increase. With the increase of tensile ratio, the crystallization of sample 1 is further improved, the crystallinity increases, the lamellar crystal thickness increases, and the scattering effect between the crystal region and the amorphous region is enhanced, so the space charge accumulation begins to decrease. In general, the space charge accumulation of sample 1 with different proportion of stretching is lower than that without stretched. For samples 2, 3, and 4, as analyzed by DSC experiment before, tensile first destroys their original crystal structure, which reduces the scattering effect of crystal region and amorphous region on carriers. Meanwhile, it can be seen from PLM experiment that when the tensile proportion is small, the distance between grains of the three samples increases significantly. These reduce the scattering of charge carriers among particles, resulting in a certain amount of space charge accumulation. When the tensile ratio reaches 20%, according to the PLM image, it can be seen that the crystal structure is relatively compact, and the distance between grains is narrowing. The scattering effect of crystal zone and amorphous zone, particle and particle on carrier is also enhanced, so the space charge accumulation is significantly reduced.

**Figure 9:**
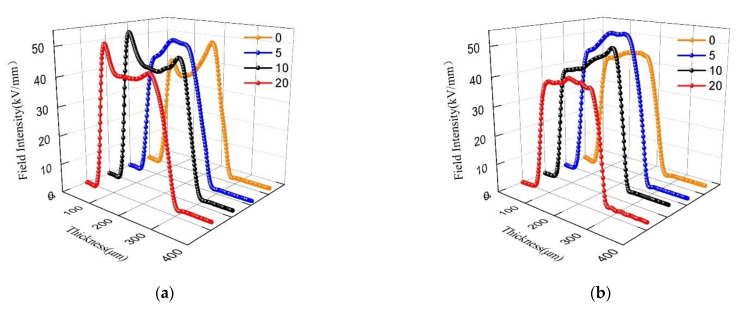

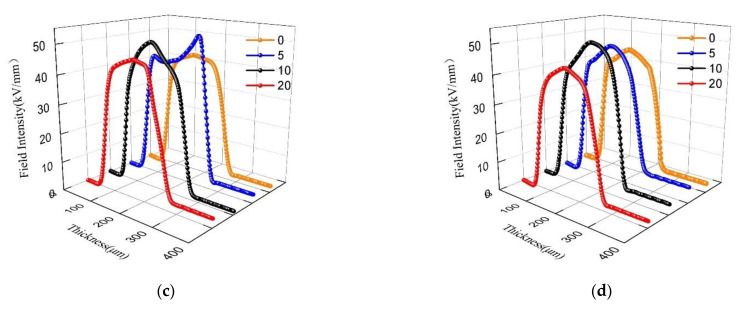
Electric field profiles in each sample with field intensity 40 kV/mm under different stretching ratio. (**a**) Sample 1; (**b**) Sample 2; (**c**) Sample 3; (**d**) Sample 4.

**Figure 10:**
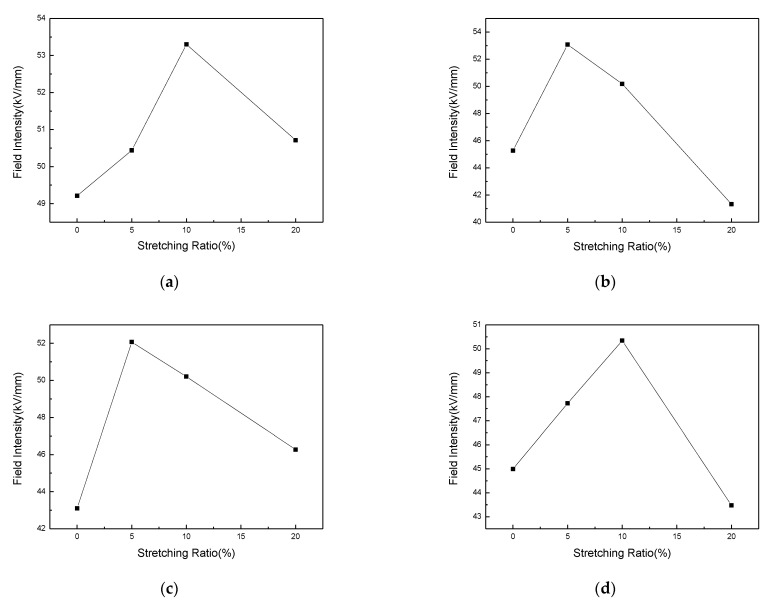
Relationship between maximum electric field and stretching ratio difference in each sample with average stress 40 kV/mm. (**a**) Sample 1; (**b**) Sample 2; (**c**) Sample 3; (**d**) Sample 4.

Figure 9 shows the variation of electric field distribution for each sample with polarization for 30 min at the field intensity of 40 kV/mm. From the figure, as the stretching ratio increases, the internal field intensity distortion for each sample shows a trend of first increasing and then decreasing. According to the experimental results in Figure 9, the relationship between the maximum distortion field intensity and the stretching ratio can be obtained, as shown in Figure 10. It can be seen from the figure that with the average field intensity of 40 kV/mm, the maximum distortion field intensity is approximately parabolically related to the stretching ratio for each sample. For sample 1, the maximum internal field intensity reaches about 49.21 kV/mm without stretched, which is about 23% higher than the average external field intensity of 40 kV/mm. When the stretching ratio reaches 10%, the maximum field intensity reaches around 53.30 kV/mm, which is 33.25% higher than the average external field intensity. With the stretching ratio continues to increase to 20%, the maximum field intensity inside LDPE begins to decrease. The observation for the other three composites shows that the maximum distortion field intensity of sample 2 and sample 3 reaches the peak value when the stretching ratio is 5%, which is 32.67% and 30.17% higher than average external field intensity, respectively. The maximum distortion field intensity in sample 4 reaches the peak value of 50.34 kV/mm when the stretching ratio is 10%.

## 5. Depolarized Space Charge Characteristics of Composites

**Figure 11:**
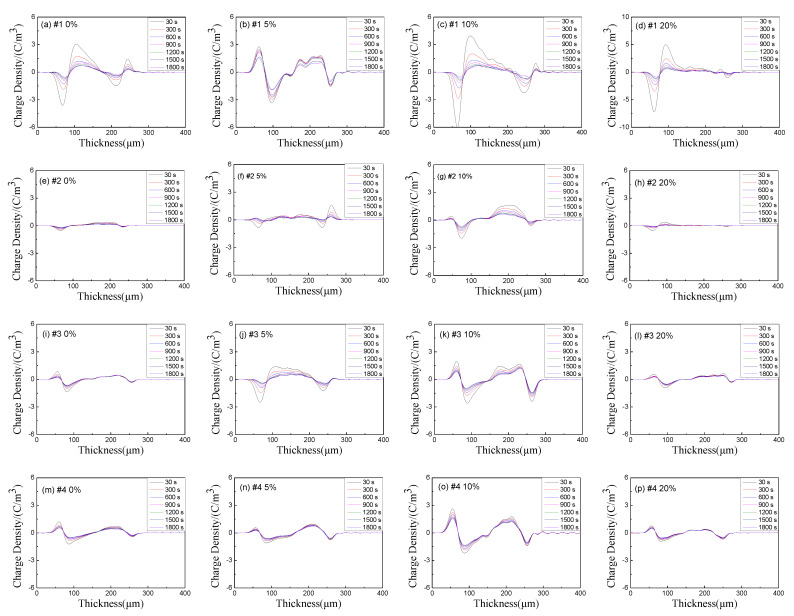
Depolarization space charge distribution curves of each sample.((**a**–**d**) are the space charge distribution curves of sample 1 under 0%, 5%, 10% and 20% tensile ratio in short circuit; (**e**–**h**) are the space charge distribution curves of sample 2 under 0%, 5%, 10% and 20% tensile ratio in short circuit; (**i**–**l**) are the space charge distribution curves of sample 3 under 0%, 5%, 10% and 20% tensile ratio in short circuit; (**m**–**p**) are the space charge distribution curves of sample 4 under 0%, 5%, 10% and 20% tensile ratio in short circuit).

After half an hour with 40 kV/mm electric field, the external electric field was removed, and the samples were short-circuited to observe their depolarization space charge distribution. The results are shown in Figure 11. It is worth noting that the limitation of the signal acquisition system makes the data collected in the early stage after short circuit unstable, so the analysis of depolarized space charge in this paper starts from 30 s. The molecular structure and morphology of polyethylene are closely related to the injection, transport and trapping of carriers. Polyethylene is composed of crystalline and amorphous regions. The residual free volume, double bonds, end groups, and interfaces between crystalline and amorphous regions will all lead to the increase of local states, which can be used as carrier traps to capture and hinder the migration of carriers and form space charges. 

According to Figure 11, it can be seen that a large number of heteropolar charges accumulate near the electrode of LDPE sample without stretched. When the stretching ratio is 5%, homopolar charges begin to accumulate near the electrode. The effects of the tensile stress on the molecular chain may deepen the trap inside the material. Deep traps capture charge will lead to homopolar charge accumulation, which will form an interface anti-electric field at the interface between the electrode and the sample, thus inhibiting the further injection of electrons or holes. Therefore, the situation in Figure 10b appears. When the stretching ratio continues to increase, it can be seen from the PLM and DSC analysis results that the crystal structure becomes further compact and regular, and the crystallization also tends to be perfect, which makes some deep traps shallow and even makes some shallow traps disappear, thus reducing the accumulation of charges with the same polarity at the electrode.

From the short-circuit curves and PLM diagrams of samples 2, 3 and 4, stretching first destroys the original compact crystal structure, which increases the internal local state and space charge accumulation of each sample. When the stretching ratio reaches 20%, the crystal structure tends to be perfect and some local state defects are eliminated, so the space charge accumulation decreases.

**Figure 12:**
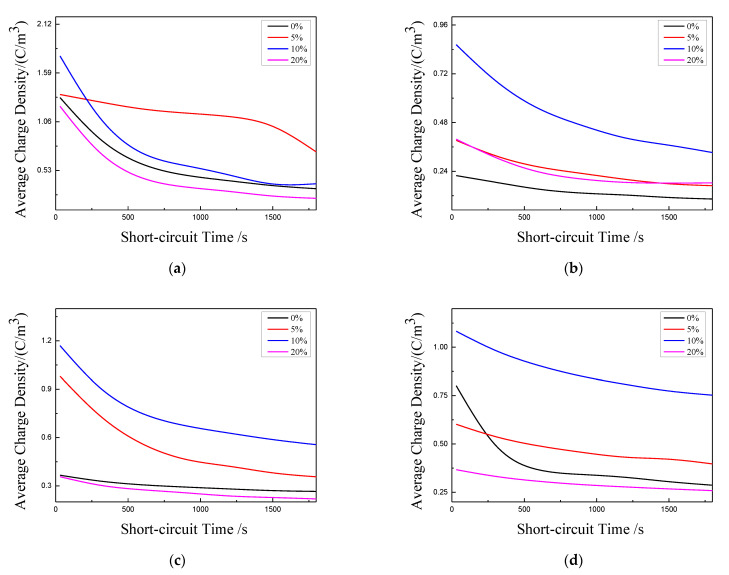
Average charge density curve of each sample. (**a**) Sample 1; (**b**) Sample 2; (**c**) Sample 3; (**d**) Sample 4.

In order to further analyze the influence of tension on space charge accumulation, according to Formula (5), the space charge density of different samples is calculated. The calculation results of average charge density of four samples under different tensile ratios during short circuit are shown in Figure 12. The average charge density for each sample shows a downward trend with the extension of short-circuit time, and the decline rate slows down and tends to be stable with the extension of short-circuit time. Additionally, with the increase of stretching ratio, the average charge density shows a trend of first increasing and then decreasing. This may be due to the tension, which makes the traps in the samples first deep and then shallow.

The charge mobility has certain reference significance for the study on related properties in polymer insulation materials and the aging diagnosis of materials. Mazzanti et al. put forward a method to estimate charge mobility, which is called apparent mobility [25]. That is to say, the mobility can be estimated according to the calculated average charge density curves under different stretching ratios. The deeper the traps in the material or the greater the trap density, the smaller the apparent mobility in the short circuit process [26]. The calculation formula of apparent charge mobility is as follows:

(6) $$\mu \left(t\right)=\frac{\varepsilon_{0}\varepsilon_{r}}{q{\left(t\right)}^{2}}\cdot \left|\frac{dq\left(t\right)}{dt}\right|$$

where $\frac{dq\left(t\right)}{dt}$ is the slope of the average charge density after short circuit with 40 kV/mm electric field, and its numerical value reflects the charge mobility, so it is taken as the absolute value in the formula; q(t) is the instantaneous value of the average charge density; $\varepsilon_{r}$ and $\varepsilon_{0}$ are relative dielectric constant and vacuum dielectric constant.

**Figure 13:**
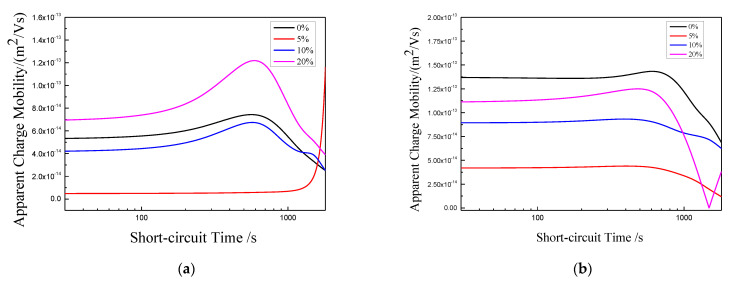

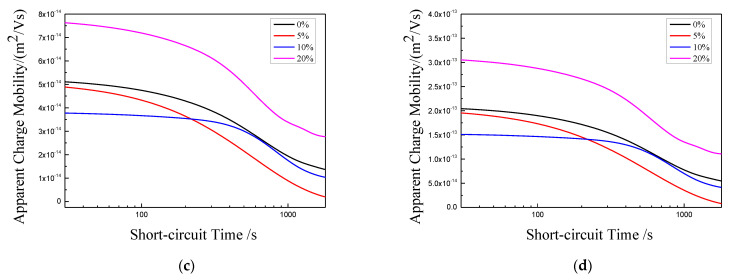
Apparent charge mobility curve of each sample. (**a**) Sample 1; (**b**) Sample 2; (**c**) Sample 3; (**d**) Sample 4.

The calculation results of apparent mobility for each sample under different stretching ratios are shown in Figure 13. For sample 1, when the stretching ratio is 5%, the charge mobility decreases rapidly, which shows that the depth of trap is really deepened by 5% stretching. The deep trap limits the carrier strongly, which makes it difficult to get rid of the bondage. This is consistent with the previous analysis of depolarization space charge distribution. At the later stage of short circuit, the charge mobility increases rapidly. To a certain extent, this reflects that the deep trap induced by 5% stretching is unstable. When the carrier concentration reaches a certain level, the trap structure may change and the carriers can be released. With the increase of stretching ratio, the crystalline structure becomes compact. The compact crystalline structure will change the localized state structure, eliminate some structural defects, and make some deep traps shallow and some shallow traps disappear in the composite material, thus the charge mobility gradually increases. 

For samples 2, 3, and 4, from the previous analysis, stretching first destroys the original compact crystal structure, which will increase the local state structure in the material. This leads to the increase of trap density, and thus make the carrier bound stronger, so the charge mobility becomes lower. With the increase of tensile ratio, the crystal structure of samples 3 and 4 becomes more compact and more compact. This reduces the internal defects in materials, resulting in the charge mobility increasing. For sample 2, when the stretching ratio is 20%, from the PLM experiment and DSC analysis results, most of its segments are still in the stage of developing into lamellae, so the lamellae formed are still thinning. The microstructure formed by this local state will further limit the charge migration, so the charge mobility will be lower than when unstretched.

## 6. Conclusions

(1) From the experimental results of PLM, it can be seen that the tensile action can make the grains of pure LDPE materials with loose crystal structure orient along the force direction, while for MMT/LDPE, SiO_2_/LDPE and MMT-SiO_2_/LDPE composites with tighter crystal structure than LDPE, the grains of samples begin to orient along the force direction when the tensile ratio reaches 10%.
(2) From the value of crystallinity and the calculation results for lamellar crystal thickness, it can be seen that, with the increase of tensile ratio, the loose molecular chains in the amorphous region of LDPE samples gradually have regular orientation arrangement, the crystallization tends to be perfect, and the crystallinity increases. As result of the addition of lamellar MMT particles, MMT/LDPE is easy to be oriented when it is stretched, and undergoes three stages: initial crystal destruction, loose molecular chain orientation, tight crystal structure. SiO_2_/LDPE and MMT-SiO_2_/LDPE composites experience three stages of initial grain squeeze, crystal structure failure and molecular chain orientation crystallization perfection, because of their tight crystal structure.
(3) From the data and analysis of space charge characteristic curve, it can be seen that, for pure LDPE samples, due to the tensile stress, the relatively loose molecular chains in LDPE samples start to orient and develop towards lamellar crystal, which will lead to the intensification of carrier scattering. At the same time, the crystallization formed by this orientation is imperfect, which will deepen the depth of traps in the samples, and the deep traps will form interface anti-electric fields, all of which leads to low space charge accumulation in the samples. With the increase of the stretching ratio, the crystal structure tends to be perfect, the internal local state structure is improved, and the trap density is reduced. When the stretching ratio reaches 20%, the internal space charge accumulation of the sample is less. For MMT/LDPE, SiO_2_/LDPE and MMT-SiO_2_/LDPE composites, stretching first destroys the compact crystal structure of these three composites. This makes the originally ordered molecular chains disordered and increases the trap density inside the sample, resulting in the accumulation for many space charges. When the stretching ratio increases, the disordered and loose molecular chains begin to orient along the force direction, the crystalline structure becomes compact, and some defects in the sample are eliminated. Meanwhile, the scattering effect of carriers between crystalline and amorphous regions, particles and particles is intensified, thus inhibiting the accumulation of space charges in the sample.

## Figures and Tables

**Table 1:** The composition and content of each composite material.

Specimen	Mass Fraction/wt%		
	SiO_2_	MMT	LDPE
1	0	0	100
2	0	1	99
3	1	0	99
4	1	1	98

**Table 2:** Melting peaks and crystallinities of all samples.

Sample 1			
Tensile Ratio	Melting Peak Temperature Tm/°C	CrystallinityXc/%	Melting Heat of Complete Crystallization/J·g^−1^
0%	110.18	31.55	92.63
5%	109.39	31.88	93.60
10%	109.68	32.27	94.74
20%	110.01	32.93	90.68
Sample 2			
0%	110.44	35.41	103.97
5%	109.98	32.16	94.42
10%	109.67	30.58	89.78
20%	109.37	35.11	103.08
Sample 3			
0%	110.36	37.69	110.66
5%	111.11	33.39	92.16
10%	109.03	29.89	87.76
20%	109.79	33.53	98.44
Sample 4			
0%	109.31	32.82	96.37
5%	109.65	32.10	94.25
10%	109.49	31.90	93.66
20%	109.53	35.38	103.88

**Table 3:** Lamellar Crystal Thickness of Each Sample.

Tensile Ratio	Lamellar Crystal Thickness (nm)			
	Sample 1	Sample 2	Sample 3	Sample 4
0%	8.56	8.63	8.61	8.33
5%	8.35	8.51	8.83	8.42
10%	8.43	8.43	8.26	8.38
20%	8.52	8.35	8.46	8.39

## Data Availability

The data presented in this study are available on request from the corresponding author.
